# Snacktivity^TM^, Giant Games and Immersive Virtual Reality Exercises: A Rapid Narrative Review of These New Physical Activity Practices among Older People Living in Nursing Homes and Long-Term Care Facilities

**DOI:** 10.3390/healthcare10101897

**Published:** 2022-09-28

**Authors:** Nounagnon Frutueux Agbangla, Marie-Philippine Séba, Frédérique Bunlon

**Affiliations:** 1 Unité de Recherche Pluridisciplinaire Sport Santé Société (URePSSS), ULR 7369, Univ. Artois, Univ. Lille, Univ. Littoral Côte d’Opale, F-62800 Lille, France; 2 Institut des Sciences du Sport-Santé de Paris (URP 3625), Université Paris Cité, F-75015 Paris, France; 3 ReSanté-Vous, F-86000 Poitiers, France

**Keywords:** physical activity, aging, Snacktivity^TM^, immersive virtual reality, giant games, nursing homes, long-term care facilities

## Abstract

In developed countries, the increasing number of older adults raises many public health challenges. Physical activity can enable healthy aging, as it is beneficial for both mental and physical health. Despite this, not all older adults engage in physical activity or have access to it. To counteract this, new physical practices such as Snacktivity^TM^, giant games and immersive virtual reality exercises are being developed. The main objective of this rapid narrative review is to summarize the effects of Snacktivity^TM^, giant games and immersive Virtual Reality (VR) exercise among older adults living in nursing homes and long-term care facilities. A scientific review was performed using the search engines PubMed, PsycInfo and Web of Science on 7 July 2022. Fifty-nine items are identified in total, and five items are included in the literature review. Synthesis of the studies shows that immersive virtual reality and giant games improve the motivation and enjoyment of older adults and are beneficial for their functional abilities. Furthermore, no interventional studies have tested the effect of Snacktivity^TM^ in older adults. This review suggests that future studies should be conducted to further explore the effects of these new physical activity practices in older adults living in nursing homes or long-term care facilities.

## 1. Introduction

Medical advances in recent decades have led to an increase in life expectancy and a consequent increase in the number of people over 65 years of age in the population of high-income countries. According to the United Nations population projections, the number of people aged 80 years and above is expected to triple, rising from 143 million in 2019 to 426 million in 2050 [1]. This substantial increase in the number of older adults has made old age a major public health problem. Indeed, aging, which is the decline or loss of adaptation with advancing age [2], induces several changes in an organism. These include decreases in basic metabolism and thermoregulation; decreases in muscle mass and strength (sarcopenia); porosity and fragility of bone tissue (osteoporosis); brain damage (neurofibrillary degeneration, senile plaques, neuronal and synaptic losses, vascular anomalies); and deterioration of sensory organs, the cardiovascular system, respiratory system, renal function, immune system, digestive system and endocrine functions [3]. All these changes, depending upon genetic predisposition and social inequalities, can lead to potentially serious pathologies, which can then lead to dependence. Dependence is the inability to perform acts of daily living unaided [4] due to the deterioration of functional capabilities. In France, 4 million older adults will lose their autonomy by 2050 [5]. Dependence, whether partial or full, especially when it is physical dependence, reduces mobility. Reduced mobility in older adults affects health and quality of life and increases social care costs [6]. Physical activity, on the contrary, maintains mobility and improves the quality of life of older adults [7].

Physical activity is part of the category of healthy physical interventions and remains a nonpharmacological treatment [8] to limit the worsening of autonomy loss in dependent older adults, whether they are at home or in nursing homes. Several studies carried out in nursing homes have shown that physical activity improves or maintains overall cognitive functioning (for review see [9]). In addition, it reduces depression and anxiety symptoms [10,11,12,13] and improves the quality of life [11,12]. Moreover, exercise has substantial benefits for the physical health of older adults and helps to maintain independence and increase longevity [14]. These effects can also be observed in older adults who have mobility problems [15]. For example, a 9-week combined program (walking and resistance exercise) not improved only endurance during walking but also leg muscle strength and balance [16] in older adults living in nursing homes. Similarly, other studies using a conventional exercise program (resistance + balance, Nordic walking, modified Sinaki exercise, walking) [17,18,19,20], exergames (Wii fit) [17] or supervised practice by videoconference (resistance) [21] have shown that exercise improves proprioception, quadriceps strength and postural balance [17], total body muscle mass, chair sit-and-reach scores [21], thoracic mobility, motor activity and motor skills [19], walking and other activities of daily life [20]. Finally, physical activity also improves the physical and functional abilities of older adults living in nursing homes [22,23,24].

Although the beneficial effects of physical activity on the health of older adults are known, it remains clear that the practice of physical activity decreases after the age of 60 years; this reduction correlates negatively with advancing age [25,26]. In France, this reduction in physical activity appears as early as age 55 in one-third of individuals [27]. In general, the level of physical activity of adults still needs to be improved. Indeed, if nine out of ten adults in France know the World Health Organization (WHO) recommendations, only two-thirds actually achieve these standards [28]. In nursing homes, the proposal of physical activity is not systematically integrated into the care of residents, and when offered, not all residents commit to it. Thus, a sedentary lifestyle and physical inactivity are highly prevalent in older adults; more women are affected as there are more women in nursing homes. Of the 728,000 older adults residing in nursing homes in Metropolitan France, three-quarters are women [29,30]. Physical activity remains beneficial for older adults despite advanced age, gender and ethnicity [31,32]. In the literature, several factors are indicated as determinants of the practice of physical activity. These include demographic, behavioral, biological, individual, social, cultural, industrial, technological, economic, and national or regional policy factors [22,33]. Specifically, in dependent older adults, the individual factor of loss of autonomy plays a predominant role in the level of practice of physical activity. Indeed, the loss of autonomy decreases the level of practice of physical activity; the reduction of physical activity is detrimental to health, and as health deteriorates, the reduction in physical activity increases further. It should also be noted that motivation and past lifestyle influence physical activity. The challenge remains in how to motivate older adults living in nursing homes to engage in a higher level of practice of physical activity.

The main objective of this rapid narrative review is to summarize the effects of Snacktivity^TM^, giant games and immersive Virtual Reality (VR) exercise among older adults living in nursing homes and long-term care facilities.

## 2. Methods

### 2.1. Search Strategy and Selecting the Evidence

**Figure 1:**
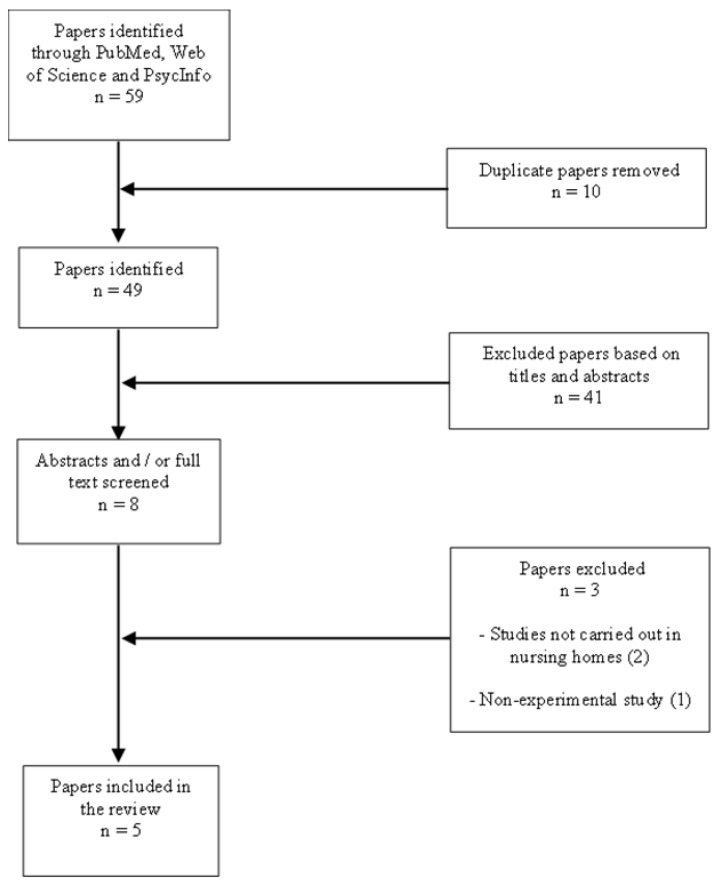
Chart flow of the selection process.

The articles were searched for by two researchers (N.F.A., M.-P.S.) in the PubMed, Web of Science and PsycINFO databases. During the search, the combination of several keywords was used. Among these combinations were ‘nursing homes’ OR ‘long term care facility’ AND ‘virtual reality exercises’, ‘nursing homes’ OR ‘long term care facility‘ AND ‘giant games exercises’, ‘Nursing Homes’ OR ‘long term care facility‘ AND ‘Snacktivity^TM^’. After the search, the title and abstract of all identified items (59 items) were scanned by two authors (M.-P.S. and N.F.A.) to remove duplicate and irrelevant studies. Then, the selected articles were scanned in their entirety to determine whether or not they should be included in the narrative review. Items included in the review are those written in English, performed in nursing homes or long-term care facilities, and those which examined the effect of new physical activity practices on psychological variables of older adults. However, items that did not use immersive VR were removed. The selection procedure for the research paper is illustrated in Figure 1.

### 2.2. Extracting the Evidence

For each article included, one author (M.-P.S.) used a standardized extraction form to extract the data. This extraction was then checked by a second author (N.F.A.) to make sure there were no errors. The standardized extraction form contained the following variables: references, design, sample, age, duration of intervention, and outcomes.

## 3. Definition of Concepts

In scientific literature, it is not uncommon to find various themes, such as physical activity, physical exercise and adapted physical activity, in studies that explore the beneficial effects of an active lifestyle versus an inactive lifestyle on health. It is, therefore, important to clarify these themes not only in terms of physical activity, physical exercise and adapted physical exercise, but also sedentary lifestyle and physical inactivity.

### 3.1. Physical Activity

Physical activity is commonly defined as ‘*Any body movement generated by the contraction of skeletal muscles that raises energy expenditure above resting metabolic rate. It is characterized by its modality, frequency, intensity, duration, and context of practice*’ [34,35] (p. 2). Other authors, considering that physical activity is a complex and multidimensional concept, have proposed another definition of activity that considers physical activity as ‘*the behavior that involves human movement, resulting in physiological attributes including increased energy expenditure and improved physical fitness*’ [36] (p. 15). Based on this definition, the authors identified four domains of physical activity: leisure activities, household activities, work- or school-related activities, and mobility activities to get from one place to another [36]. In its guidelines on physical activity and sedentary lifestyle, WHO has issued recommendations for different categories of the population. According to these recommendations, people over 65 years of age should perform aerobic activities at least three times a week (150 to 300 min of moderate intensity, or 75 to 150 min of sustained intensity), muscle strengthening exercises two times per week (moderate or sustained intensity) and balance exercises three times per week [37]. Achieving these WHO recommendations requires some organization and planning, but ultimately leads to physical exercise.

### 3.2. Physical Exercise and Adapted Physical Activity

Physical exercise is a ‘*subcategory of physical activity that is planned, structured, repetitive, and that favors physical fitness maintenance or development*’ [34,35] (p. 2). However, because older people living in nursing homes often face a loss of autonomy, physical activity needs to be adapted for these individuals to benefit from physical activity. Adapted physical activity is defined as ‘*any movement, physical activity and sport, essentially based on the aptitudes and motivations of people with specific nreeeds that prevent them from practicing in ordinary conditions*’ [38] (p. 628). This definition was later completed in 2021 by the French Society of Adapted Physical Activity, and adapted physical activity was redefined as ‘*the scientific and professional field of physical activity aimed at any person who does not or cannot practice a physical activity or sport under ordinary conditions and who has specific needs for health, social participation or inclusion due to an illness, a functional limitation, a deficiency, a vulnerability, a situation of disability, exclusion, inactivity or sedentary behavior*’ [39] (p. 1).

### 3.3. Physical Inactivity and Sedentary Behavior

Physical inactivity refers to the failure to meet official guidelines or recommendations for regular physical activity [35,40]. It has serious consequences on health. Indeed, physical inactivity is a factor leading to the appearance of several non-transmissible diseases such as cancer, cardiovascular diseases, diabetes, depression, etc. [40,41,42]. Nevertheless, although physical inactivity has become the leading cause of death in Western countries, it is preventable [43]. With regard to sedentary behavior, it is defined as ‘*Any waking behaviors characterized by an energy expenditure ≤1.5 METs, while in a sitting, reclining, or lying posture*’ [35,44]. Sedentary behavior is more prevalent in older adults [45], and therefore influences their health. To avoid the harmful effects of sedentary behavior in older adults, it is important to do everything possible to reduce their sedentary time. 

## 4. Results

This section describes the main features of the new physical practices. The following paragraphs present the five studies selected with a description of the types of study design, experimental and control groups, intervention designs, and the types of exercises included. Table 1 sums up the main characteristics of the selected studies.

### 4.1. Study and Interventions Characteristics

Five items that used either immersive VR (n = 3) or giant games (n = 2) were included in this narrative review. Immersive VR systems can themselves be perceived as exergames or serious games [46]. It should be specified that immersive VR systems are those which, with regard to the visual interface, do not allow the user to see their surrounding physical environment, but only the virtual environment, including cave automatic virtual environments or headsets [47]. Regarding giant games, they consist of a play mat that allows players to perform strength, flexibility, balance and endurance activities [48].

Several experimental designs, such as quasi-experimental studies [49,50], within-subject study with pre- and post-intervention measures [51], prospective crossover proof of concept study [52] and pilot interventional study [48], have been used in the items. Of all the five items, three did not include control groups in their experimental design [49,51,52]. Sixty-seven older adults (35 women, 32 men) aged between 70 to 86.8 years were included in the experimental groups. The control group included 21 older adults (14 women, 7 men) aged between 84.5 and 89.9 years.

The immersive VR interventions [49,51,52] lasted between 1 h and 12 weeks. During each session, participants performed two to five sets of 20 or 30 min of exercise (cycling or leap motion blocks, slum ball VR tournament, VR sports—basketball, VR sports—soccer; motion: neck rotation, reaching forward and straight, reaching forward and across the body, rowing; or activity in farm game: watching a butterfly, lifting a box of apples, sorting fruits in buckets, rowing a rowboat) per week. The giant games interventions [48,50] lasted one month and consisted of strength, flexibility, balance and endurance exercises. Each session lasted 30–60 min and participants were encouraged to practice as much as possible on their own outside the supervised sessions. The studies’ characteristics, interventions and measured main variables are summarized in Table 1.

**Table 1:** Characteristics of the studies included in the review.

References	Study Design	Participants	Age	Duration of the Intervention	Main Measures
Buckinx et al., 2020 [48]	Pilot interventional study	Study group: 11 (4 women) Control group: 10 (6 women)	Study group: 70 years Control group: 84.5 years	1 month of intervention	Cognitive status (MMSE), quality of life (EuroQol 5 dimensions), motivation for physical activity (BREQ-2), physical capacity (Tinetti test, SPPB, TUG, grip strength, isometric strength of the lower limb muscles and quantitative gait analysis)
Chen et al., 2021 [49]	Quasi experimental study	30 (20 women)	74.57 ± 12.83 years	2 × 30 min for 12 weeks	Dominant handgrip strength, walking speed and appendicular skeletal muscle mass index
Eisapour et al., 2020 [51]	Within-subject study with pre- and post-intervention measures.	6 (5 women)	86.8 ± 6.2 years	5 × 20 min sessions per week for 3 weeks	Qualitative and quantitative measures, including reaching distance, distance traversed, engagement, interest, easiness, comfort, and level of effort.
Loggia et al., 2021 [52]	Prospective crossover proof-of-concept study	10 men	75.3 ± 8.5 years	2 × 30 min of cycling.	Cycling distance, pedaling duration, average speed, mean pedaling cadence and the modified Borg rating of perceived exertion scale
Mouton et al., 2017 [50]	Quasi-experimental longitudinal study	Study group: 10 (6 women) Control group: 11 (8 women)	Study group: 82.5 ± 6.3 years; Control group: 89.9 ± 3.1 years	1 month of intervention and 3 months follow-up	Expenditure/day with ActiGraph, cognitive status (MMSE), quality of life (EuroQol 5 dimensions), motivation for physical activity (BREQ-2), gait and balance (Tinetti and SPPB), functional mobility (TUG), and the muscular isometric strength of the lower limb muscles

BREQ-2 = Behavioral Regulation in Exercise Questionnaire-2; MMSE = Mini Mental State Examination; SPPB = Short Physical Performance Battery; TUG = Timed Up and Go.

### 4.2. Effects of New Physical Activity Practices among Older People Living in Nursing Homes or Long-Term Care Facilities

The results of the few studies included in this review show that older adults improve their physical performance (cycling distance and duration) when they exercise with immersive VR compared to exercising without immersive VR [52]. In addition, the same authors found that participants preferred to repeat the cycling sessions with virtual reality rather than without. Another important result was that in older adults with dementia, immersive VR and human-guided exercises had the same effects on participants’ subjective responses, motion (reaching distance, speed and distance) and fitness parameters (head-to-wall, reach downward, reach upward, grip strength, shoulder flexibility) [51]. Finally, immersive VR based on progressive resistance training was found to significantly improve handgrip strength and walking speed [49]. Regarding the giant games, the results show that after the first month of intervention, the older adults in the experimental group displayed an increase in the number of steps per day. This increase in steps is also observed after the three-month follow-up. The same pattern of increase is also observed in energy expenditure. Apart from the number of steps and energy expenditure, participants’ quality of life, balance, gait, and ankle strength improved after the three-month follow-up. [50]. Later, a new study confirmed and completed the effects of the giant games on Tinetti scores, TUG, quality of life, as well as on knee extensor isometric strength, grip strength, symmetry of steps and intrinsic motivation [48]. 

Finally, we found that no quantitative study have tested the effect of Snacktivity^TM^ on the psychological or physiological parameters in older adults. The few studies that do exist in the literature [53,54,55] are qualitative studies or commentaries.

## 5. Discussion

The effects of immersive VR and giant games on the physical abilities of older adults living in nursing homes or long-term care facilities are not surprising. Indeed, whether considering immersive VR or giant games, the interventions used in the different studies [49,51,52] proposed aerobic, strength, flexibility and balance exercises for these older adults. However, these types of exercises, specifically aerobic and resistance exercises, are known to attenuate the loss of muscle mass during aging through the activation of anabolic signaling pathways, and improve cardiorespiratory fitness through the improvement of cardiovascular function and oxidative capacity, respectively [56]. Thus, these physiological adaptations would be the basis for the improvements observed in older adults’ strength, walking, balance, and functional capacities. Another observed effect of immersive VR and giant games is the effect on motivation and pleasure. Indeed, older adults increase their motivation and enjoyment by exercising in giant games or using immersive VR. This increase is consistent with other studies that have used virtual reality [57,58] and can be explained by the fact that immersive VR reduces the sensation of pain during exercise [59].

Another important finding of our review is the lack of studies that use Snacktivity^TM^ to explore its effects on the psychological and physiological parameters of older adults living in nursing homes or long-term care facilities. Indeed, Snacktivity^TM^ works to motivate and help individuals be more physically active throughout the day by providing small (2–5 min) durations of moderate to vigorous intensity physical exercise (walking and chatting, preferably taking the stairs, doing push-ups against the stairs, etc.) on a regular basis to reach the recommended 150 min/week of physical activity [53]. The lack of studies on this topic could be explained by the fact that this concept is very new in the literature. However, this concept appears to be interesting in many ways. Indeed, inactive people believe that Snacktivity^TM^ could help them increase their level of physical activity [54] because it is easy to accomplish [55]. It is clear that despite the beneficial effects of these new practices, very few studies have been performed in nursing homes or long-term care facilities. It would, therefore, be interesting for researchers to explore these new practices, especially by observing their effects on users’ executive functions. For example, Snacktivity^TM^ could be combined with immersive VR or giant games to test its effect, in an interventional protocol, on executive functions (inhibition, working memory updating, cognitive flexibility and dual-tasking) that decline during aging.

At last, this review has some limitations. Indeed, the search for articles was conducted in three databases (PubMed, PsycInfo and Web of Science) while focusing on articles published in English. This strategy could lead to the omission of articles published in other languages and in other databases. In addition, the studies included in this review used small sample sizes, sometimes without control groups. Thus, the beneficial effects of the new practices highlighted in this review should be considered with caution and should be confirmed in future studies with a large sample size.

## 6. Conclusions

Synthesis of the studies included in the current review suggests that new physical practices such as immersive VR, giant games and Snacktivity^TM^ could facilitate the promotion of physical activity. These new physical practices may increase not only the motivation and enjoyment of older people, but also to enhance their functional capacities. Moreover, these new physical practices should be used more in these facilities (nursing homes or long-term care facilities) with the sole objective of helping the older adults practice at their best while experiencing pleasure. Above all, the effects of these new physical practices should be further explored in nursing homes and long-term care facilities.

## Data Availability

Not applicable.
